# Genome-wide association study provided insights into the polled phenotype and polled intersex syndrome (PIS) in goats

**DOI:** 10.1186/s12864-024-10568-9

**Published:** 2024-07-02

**Authors:** Fuhong Zhang, Qingqing Liu, Ping Gong, Yaling Wang, Chenbo Shi, Lu Zhu, Jianqing Zhao, Weiwei Yao, Jun Luo

**Affiliations:** 1 Key Laboratory of Animal Genetics, Breeding and Reproduction of Shaanxi Province, College of Animal Science and Technology, Northwest A&F University, Yangling, 712100 P. R. China; 2 Institute of Animal Husbandry Quality Standards, Xinjiang Academy of Animal Sciences, Urumchi, 830000 P. R. China

**Keywords:** Goat, Whole-genome sequencing, GWAS, Horn, SNP

## Abstract

**Background:**

Breeding polled goats is a welfare-friendly approach for horn removal in comparison to invasive methods. To gain a comprehensive understanding of the genetic basis underlying polledness in goats, we conducted whole-genome sequencing of 106 Xinong Saanen dairy goats, including 33 horned individuals, 70 polled individuals, and 3 polled intersexuality syndrome (PIS) individuals.

**Methods:**

The present study employed a genome-wide association study (GWAS) and linkage disequilibrium (LD) analysis to precisely map the genetic locus underlying the polled phenotype in goats.

**Results:**

The analysis conducted in our study revealed a total of 320 genome-wide significant single nucleotide polymorphisms (SNPs) associated with the horned/polled phenotype in goats. These SNPs exhibited two distinct peaks on chromosome 1, spanning from 128,817,052 to 133,005,441 bp and from 150,336,143 to 150,808,639 bp. The present study identified three genome-wide significant SNPs, namely Chr1:129789816, Chr1:129791507, and Chr1:129791577, as potential markers of PIS-affected goats. The results of our LD analysis suggested a potential association between *MRPS22* and infertile intersex individuals, as well as a potential association between *ERG* and the polled trait in goats.

**Conclusion:**

We have successfully identified three marker SNPs closely linked to PIS, as well as several candidate genes associated with the polled trait in goats. These results may contribute to the development of SNP chips for early prediction of PIS in goats, thereby facilitating breeding programs aimed at producing fertile herds with polled traits.

**Supplementary Information:**

The online version contains supplementary material available at 10.1186/s12864-024-10568-9.

## Introduction

Horns are cranial appendages unique to ruminant animals [1]. The presence of horns is crucial for self-defense [2, 3] and serves as a prominent sexual trait in intra-male competition [4]. However, the utilization of horns in inter-animal fights poses risks to the herd’s welfare and complicates management and transportation processes, consequently leading to increased economic burdens [5]. Therefore, exfoliation is increasingly gaining popularity in modern animal husbandry. Undoubtedly, breeding polled animals offers two primary advantages. Firstly, it enhances animal welfare by minimizing injuries resulting from inter-animal fighting and avoiding the significant distress caused by conventional dehorning methods during the early stages [6]. Secondly, it protects animal caretakers, particularly farmers and all people in contact with animals [7]. Although the polled trait follows a dominant inheritance pattern, selective breeding for polledness poses challenges due to the risk of inbreeding and loss of genetic variance, as polled animals do not have the genetic level achieved as horned populations [8]. With the development of science and technology, the application of modern animal breeding techniques, such as high-density genotyping and genome resequencing, will facilitate the resolution of this biological issue.

In recent years, extensive investigations have been conducted on the genetic basis underlying the horned and polled phenotypes in cows, sheep, and goats. It has been shown that the molecular genetic causes for polled phenotypes vary due to distinct genetic backgrounds across different species. In cows, the polled individuals are inherited as an autosomal monogenic dominant trait, where the *P* allele (hornless) exhibits dominance over the *p* allele (horned) [9]. Although the polled locus has been reported to be located on the proximal end of bovine chromosome 1 [10], the causal genetic variants linked to the polled phenotypes are distinct in cows from diverse breeds. For instance, a total of four distinct genetic variants have been identified to be linked to the polled phenotypes in different European breeds [11, 12]. In sheep, the researchers found that the insertion of a nucleotide sequence of about 1.78 kb in chromosome 10 was the factor leading to the emergence of hornless sheep [13, 14]. However, the insertion alone does not fully account for the polled phenotype observed in diverse sheep breeds [15]. In goats, the inheritance pattern of polled phenotypes resembles the monogenic autosomal dominant mode observed in cows. The absence of horn growth in goats is attributed to a complex structural genetic variant, characterized by the fusion of a large duplicated segment measuring 480 kb, located at position 150,334,286–150,818,098 bp on chromosome 1, into the deleted region spanning approximately 11.7 kb situated around 129 Mb on chromosome 1 [16, 17]. However, the specific elements of these intricate structural variations that contribute to the absence of horns remain indeterminate.

Polled intersexuality syndrome (PIS) goats are occasionally observed within polled flocks. The dominant inheritance of the polled phenotype is mainly linked to recessive intersexuality in goats [18]. Homozygous polled females typically exhibit infertility as intersex individuals, while homozygous polled males do not display any signs of intersexuality. Previous studies have demonstrated that a deletion spanning 11.7 Kb, located at approximately 129 Mb on chromosome 1, is postulated to underlie the etiology of PIS-affected Saanen goats [16]. The most significant observation is that a duplicated sequence of approximately 480 Kb, which was originally located at the genomic position of around 150.5 Mb on chromosome 1, underwent an inverse insertion event into the PIS locus [9, 16]. Although phenotypes are believed to arise from gene copy number amplification, PIS often cannot be fully explained by alterations in gene dosage alone [19].

Compared to low-density SNP microarrays, the utilization of whole genome sequencing (WGS) data enables precise localization of association signals in genome-wide association studies (GWAS), thereby significantly enhancing the likelihood of identifying candidate genes responsible for causal mutations affecting complex traits in animal and plant [20–22]. In this work, we conducted a GWAS to identify the genetic variants and key candidate genes associated with the polled phenotype in Xinong Saanen dairy goats. Our findings have unveiled that two genomic regions located on chromosome 1 play a pivotal role in determining the polled phenotypes observed in goats. The identification of key candidate genes and SNPs associated with PIS-affected goats enables the development of SNP chips for early prediction of infertility intersexuality, thereby facilitating advancements in the field.

## Methods

### Animals

In this study, a total of 106 Xinong Saanen dairy goats were randomly selected from the experimental farm situated at Northwest A&F University in Yangling, Shaanxi Province, China. Informed consent was obtained from the owner. The samples comprised 33 horned individuals, 70 polled individuals, and 3 PIS individuals (Table S1). The goats utilized in this study were born from 2019 to 2022. The animals were uniformly managed and provided with a mixed diet comprising corn, soybean meal, bran, rapeseed meal, and a mineral-vitamin premix. Additionally, the reproductive strategy employed was consistent. The types of horn phenotypes have been well documented. Three PIS goats were identified based on their genital phenotype (Figure S1). The experimental protocol, including blood sampling and PIS goat identification, was approved by the Animal Ethical and Welfare Committee of the College of Animal Science and Technology, Northwest A&F University, Yangling, China (protocol number DK2022008).

### Samples collection, DNA extraction, and whole-genome sequencing

Blood samples were obtained via jugular venipuncture from selected goats. The DNA sample was extracted from blood and quantified using established experimental protocols to ensure accuracy and precision. Subsequently, the DNA samples were randomly fragmented into 350 bp fragments, followed by library construction through a series of sequential steps including end repair, polyA tailing, purification, PCR amplification, and so forth. Whole-genomic sequencing was conducted using the Illumina NovaSeq 6000 platform (Beijing, China).

### Short-read alignment and variant calling

The Trimmomatic software (version 0.38) was employed for the removal of adaptor sequences and elimination of low-quality reads [23]. The high-quality reads were then aligned to the goat reference genome (Accession NO, GCA_001704415.1) using BWA software (version 0.7.17) with default settings [24]. The Picard software (v2.10.6) (http://broadinstitute.github.io/picard/) was utilized for the removal of duplicate reads and subsequently sort the bam files. The HaplotypeCaller tool in GATK (v3.8-1-0) was employed with default parameters to detect variants for each accession [25]. Subsequently, the CombineGVCFs and GenotypeGVCFs tools in GATK were utilized to call population-level variants.

### Quality control and annotation for genotype data

To obtain high-quality variants, the raw variant calls (SNPs and Indels) were filtered based on quality and depth using VCFtools software with the following parameters: ‘--minQ 100 --minDP 3 --maxDP 100 --min-meanDP 3’ [26]. The biallelic SNPs used for subsequent analyses were obtained by applying a variant site filter using the PLINK software with the following parameters: ‘--geno 0.2 (excluding genotypes with more than 20% missing data), --maf 0.05 (removing variants with a minor allele frequency less than 0.05), and --biallelic-only’ [27]. In addition, the identified SNPs were annotated using the ANNOVAR software [28].

### Population structure

In order to understand population structure and help control the false positive results of GWAS, we performed a population genetics analysis. The biallelic SNPs were filtered based on linkage disequilibrium (LD) and retained unlinked SNPS for population structure analysis and principal component analysis (PCA) using the PLINK software with the following parameters: ‘--indep-pairwise 50 10 0.2 (filtering out SNPs with a correlation coefficient greater than 0.2 in sliding windows of 50 Kb with a 10 SNP step)’. The genotypes of selected sites were extracted from the biallelic SNP vcf file and subsequently transformed into the ‘admixture’ format using PLINK. The genotypes of 106 animals were utilized for three replicate runs conducted with K = 2–5, where K represents the assumed number of subpopulations. For principal component analysis (PCA), we initially utilized the PLINK software to convert the genotype files in vcf format into *.bed, *.bim, and *.fam files (--make-bed). Subsequently, the genetic relationship matrix and PCA were calculated using gcta64 software with the parameters: ‘--make-grm’ and ‘--pca’, respectively [29].

### Linkage disequilibrium analysis

To assess population characteristics, we performed linkage disequilibrium decay analysis on all samples, horned population, and polled population, respectively. Initially, PLINK software was employed to convert vcf files into ped and map formats (--recode ped). Subsequently, the following parameters were provided for LD analysis in PLINK: ‘--map, --ped, --noweb, --allow-no-sex, --allow-extra-chr, --missing-genotype 0, --maf 0.05, --r^2^, --ld-window 999999, --ld-window-kb 1000, and --ld-window-r^2^ 0’. The results are summarized using the script decay_chrom.pl. After identifying a significant correlation interval through GWAS, we further utilized Local LD haplotype block maps to determine the presence of an LD relationship between the significantly associated single nucleotide polymorphisms (SNPs) and the target genes. The analysis of LD blocks was conducted using LDBlockShow [30].

### Genome-wide association analysis

GWAS was performed using a mixed linear model (MLM) implemented in TASSEL software [31]. Genotypes (SNPs) and population structure (Q) were considered as fixed effects while accounting for kinship (K) as random effects in the model. The Q matrix was derived from the results of population structure analysis, and the K matrix was calculated using gcta64 software [29]. The genotype data was first sorted using the run_pipeline.pl script. Subsequently, the following command was executed: “perl run_pipeline.pl -fork1 -vcf sort.vcf -fork2 -r trait.txt -fork3 -q Q.txt -excludeLastTrait -fork4 -k K.txt -combine5 -input1 -input2 -input3 -intersect -combine6 -input5 -input4 -mlm -mlmVarCompEst P3D -mlmCompressionLevel None -runfork1 -runfork2 -runfork3”. Manhattan plots were generated using the CMplot package (https://github.com/YinLiLin/R-CMplot) within R v4.1.0.

## Results

### Sequencing variants and population stratification

The whole-genome sequencing (WGS) data were aligned to the goat reference genome (Accession NO, GCA_001704415.1), which was assembled using genomic DNA from a horned San Clemente goat. This alignment resulted in a mean genome coverage of 99.8% and an average sequencing depth of 13.21× (Table S1). After filtering out loci with deletion rates exceeding 20% and minor allele frequencies below 0.05, we identified a total of 15,944,055 biallelic single nucleotide polymorphisms (SNPs) and 1,391,598 insertions/deletions (indels) in the cohort comprising 106 individuals.

**Fig. 1:**
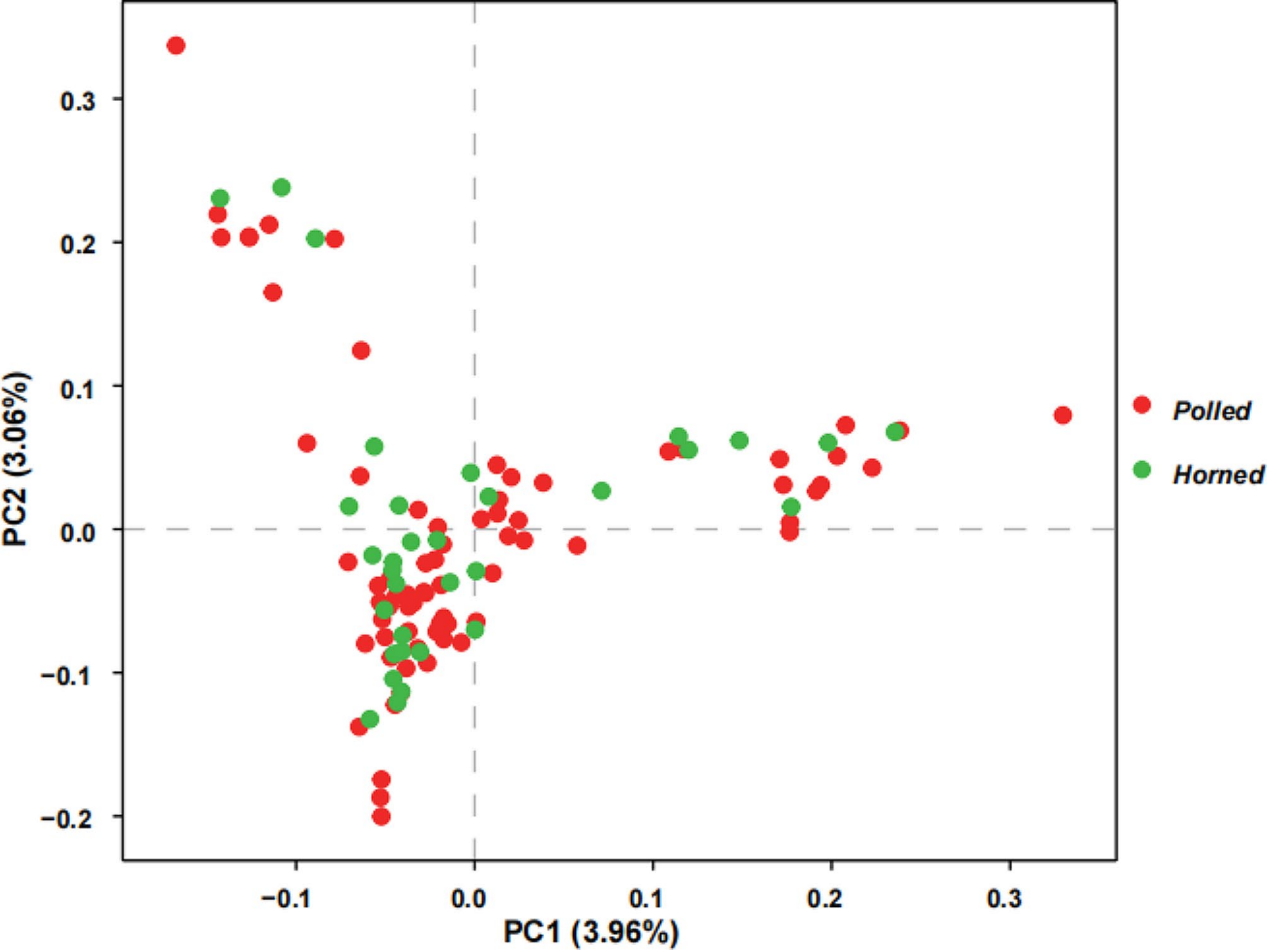
PCA plot showing the population structure of 106 sampled goats based on the identified biallelic SNPs. The red and green circles represented polled and horned goats, respectively

After filtering out SNPs with a correlation coefficient greater than 0.2 (r^2^ > 0.2) in sliding windows of 50 Kb with a 10 SNP step, we performed a population structure analysis. PCA revealed a slight population stratification among the 106 individuals, potentially suggesting familial relatedness (Fig. 1). However, the cumulative proportion of genetic variance explained by the first two principal components was relatively modest, accounting for only 3.96% and 3.06%, respectively. Similar findings were obtained from population structure analysis (Figure S2).

### Genome-wide association study

**Fig. 2:**
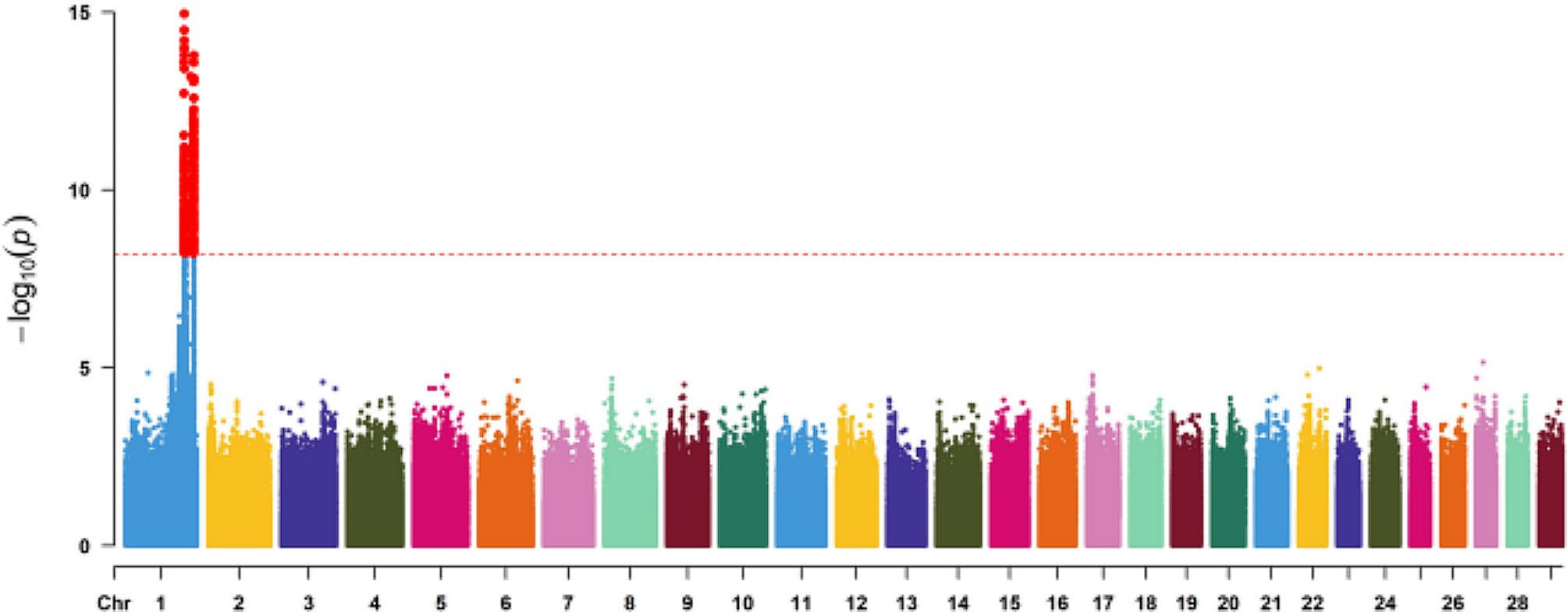
Manhattan plots depicting the results of a genome-wide association study for single nucleotide polymorphisms located on autosomes 1–29 associated with the polled traits. The chromosomes are depicted in distinct colors and presented separately. The horizontal red dashed line denotes the genome-wide Bonferroni-corrected significance threshold

After undergoing rigorous quality control and passing the Hardy-Weinberg test, we successfully identified a set of 15,059,449 biallelic SNPs that are suitable for subsequent GWAS. To account for potential confounding factors related to population structure, we performed a robust mixed linear model (MLM) association analysis using the 106 goats. The threshold value was calculated using the Bonferroni multiple test correction (0.05 divided by the total number of SNAs, at a 5% level of significance) [32]. A total of 320 significant SNPs (Bonferroni-corrected *P* < 0.05, −log_10_*P* = 8.17) associated with the horned and polled phenotypes were identified, all of which were located on chromosome 1 (Table S2). The Manhattan plot revealed two association signals (Fig. 2). The first peak of association spanned from 128,817,052 to 133,005,441 bp on chromosome 1 and consisted of 189 genome-wide significant SNPs. Among these SNPs, the top ten most associated ones (-lg_10_*P* > 12.71) were located at positions ranging from 129,410,163 to 129,791,577 bp. The second association signal was identified at the genomic coordinates of 150,336,143 – 150,808,639 bp on chromosome 1. This region encompassed a total of 131 genome-wide significant SNPs, with the top 15 SNPs (−lg_10_*P* > 12.01) being most strongly associated within the range of 150,376,727 – 150,744,739 bp (Table S2).

### Characterization of the genome-wide significant SNPs

The variant annotation revealed that out of a total of 320 genome-wide significant SNPs, 232 were located in intergenic regions, 48 in intronic regions, 39 in ncRNA intronic regions, and one in gene downstream regions (Table S3). The first associated region comprises nine genes, namely *NMNAT3*, *RBP2*, *MRPS22*, *PIK3CB*, *ESYT3*, *MRAS*, *SOX14*, *PPP2R3A*, and *FOXL2* (ENSCHIG00000026523), and six ncRNAs (ENSCHIG00000006793, ENSCHIG00000006799, ENSCHIG00000006802, ENSCHIG0000001813, U1-201, and U6). The second associated region encompasses two genes (*ERG* and *KCNJ15*), one snRNA (U6), and one small nucleolar RNA (SNORA70).

### Identification and characterization of variants associated with the horned and polled phenotypes

**Fig. 3:**
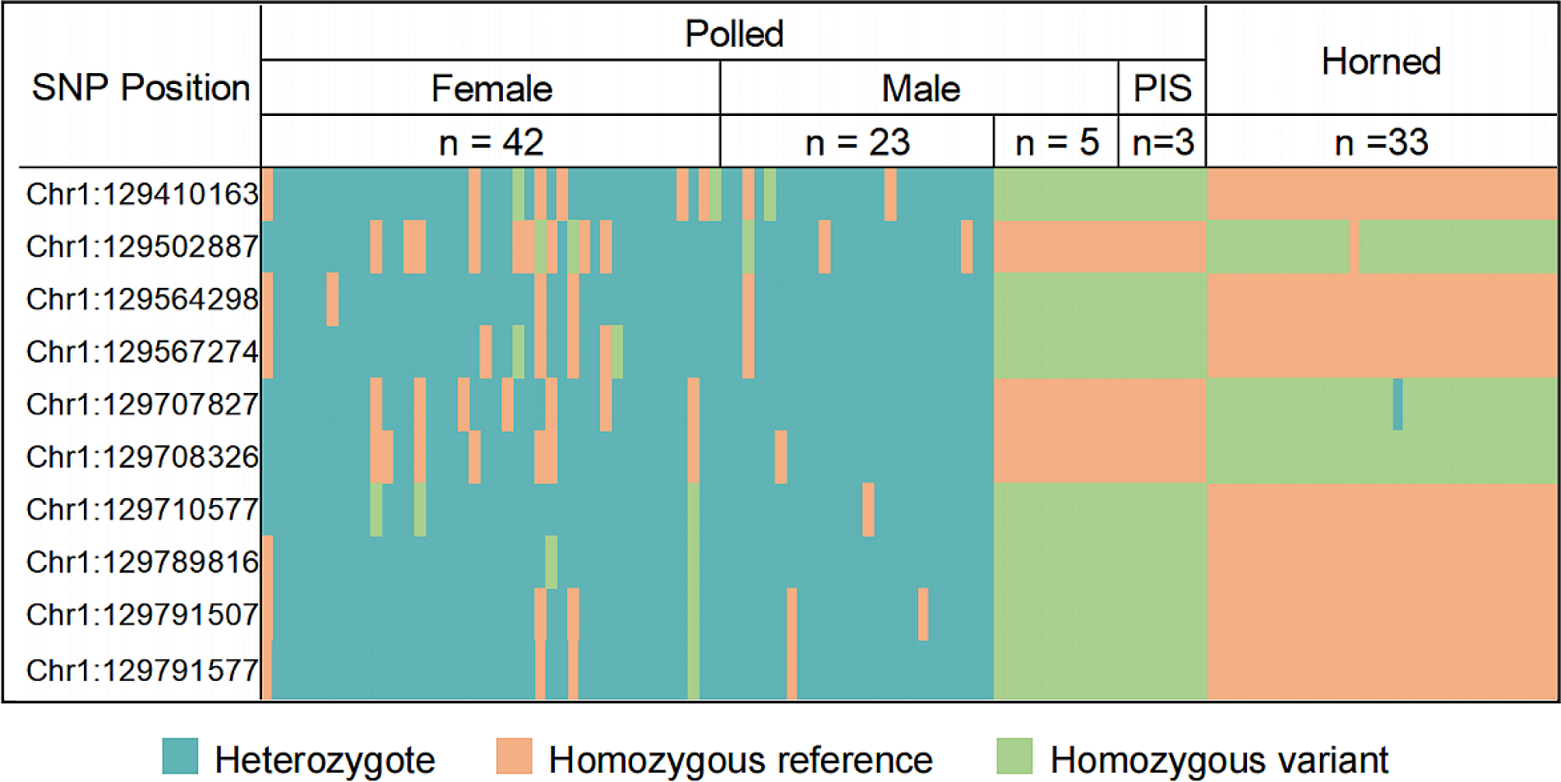
Summary of the genotype distributions for the top 10 genome-wide significantly associated SNPs in the first identified genomic region among horned, polled, and PIS goats. The three PIS goats exhibited homozygosity at three loci with reference alleles, while seven loci showed variant alleles - a contrasting pattern to that observed in horned goats. Notably, the same haplotype as the intersex goat was also identified in five polled male goats

**Fig. 4:**
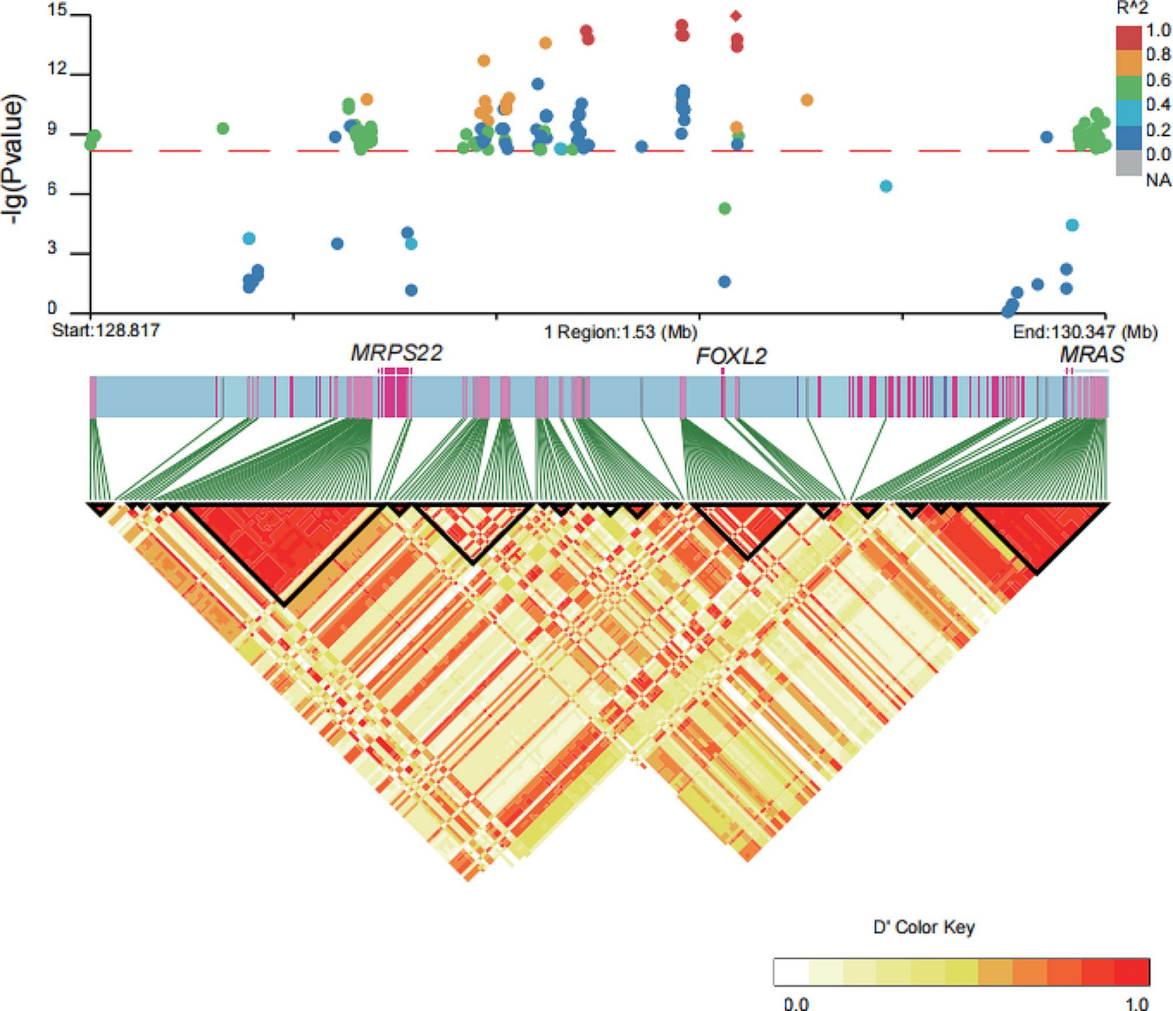
Linkage disequilibrium (LD) heatmap depicts the regions surrounding the strong peaks of the first significant association region. The genes *MRPS22*, *FOXL2*, and *MRAS* exhibit strong linkage disequilibrium relationships with the significant genome-wide association SNPs identified in the first association signal

The significantly associated SNPs of the first region predominantly localize within the range of 129,000,000 to 130,005,000 bp on chromosome 1. According to variant annotation, the top 10 genome-wide most significantly associated SNPs in the first significant signal were all located within intergenic regions. Among these SNPs, we observed predominant heterozygosity in almost all polled goats, while all 33 horned goats exhibited homozygosity and carried reference alleles at 7 loci along with variant alleles at 3 loci. All 3 PIS goats displayed homozygosity with 3 loci displaying reference alleles and 7 loci exhibiting variant alleles - an opposite pattern compared to that observed in horned goats. Interestingly, the identical haplotype to that of the intersex goat was also found in 5 polled male goats (Fig. 3). Previous studies have demonstrated a dominant inheritance pattern in polled goats, wherein homozygous polled females (XX) are associated with infertile intersex individuals, while homozygous polled males do not exhibit intersexuality [18]. The genotypes of 7 SNPs (Chr1:129410163, Chr1:129564298, Chr1:129567274, Chr1:129710577, Chr1:129789816, Chr1:129791507, and Chr1:129791577) in our results were consistent with the aforementioned research conclusions, suggesting their potential association with the polled phenotype and intersexuality, thus indicating their suitability as marker SNPs. To screen tag SNPs and determine the potential candidate genes in this specific genomic region, we conducted LD block analysis using LDBlockShow. We identified a total of 23 LD blocks (Table S4). 3 out of 7 marker SNPs (including the leading associated SNP) exhibited a strong linkage disequilibrium with the *FOXL2* gene (Fig. 4), which is widely recognized as a pivotal determinant of female sex development in mammals [33]. Therefore, 3 SNPs, Chr1:129789816, Chr1:129791507, and Chr1:129791577, can be regarded as potential indicators of the PIS-affected goats.

In the largest LD blocks, we observed a strong LD between 3 exonic SNPS in gene *MRPS22* (Chr1:129294870, Chr1:129300519, and Chr1:129300647) and 39 significant genome-wide association SNPs (Fig. 4 and Table S4). Among the 39 SNPs examined, we observed that all three PIS goats exhibited homozygosity and carried variant alleles, while not all 33 horned goats displayed homozygosity and carried reference alleles. According to the variant annotation, we observed two synonymous mutations (Chr1:129294870, c.G > A, p.T150T and Chr1:129300647, c.G > C, p.T3T) and one nonsynonymous mutation (Chr1:129300519, c.G > A, p.A46V) in the exonic regions of *MRPS22*, but they did not show significant associations. To further determine whether there is a causative relationship between *MRPS22* and polled intersex syndrome, we performed the LD analysis on the horned population and the polled population, respectively. The results revealed a significant LD between the 39 genome-wide significant association SNPs and *MRPS22*, forming an independent LD block in horned goats, while no such LD block was observed in polled goats. These findings showed that *MRPS22* may play a role in the development of PIS-affected goats. Additionally, 29 genome-wide significant association SNPs located in intronic regions of *MRAS* were found to be enriched in the second-largest LD blocks (Fig. 4 and Figure S3). However, *MRAS* exhibited 4 synonymous mutations (Chr1:130287936, c.C > T, p.T198T, Chr1:130287942, c.C > A, p.R196R, Chr1:130296909, c.G > A, p.H102H, and Chr1:130296915, c.G > A, p.F100F) in the exonic regions.

**Fig. 5:**
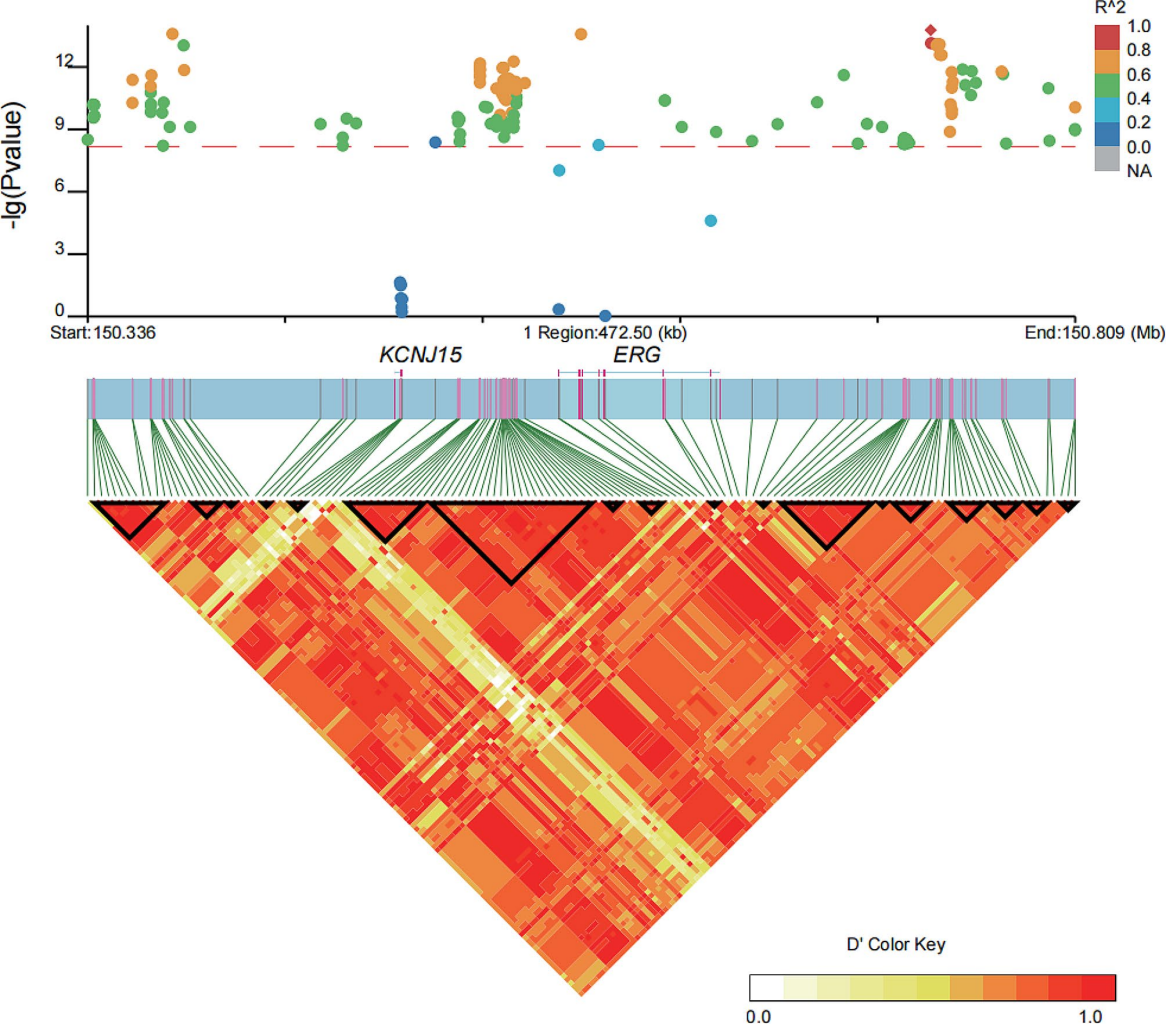
Linkage disequilibrium (LD) heatmap depicts the regions surrounding the strong peaks of the second significant association region. The genes *ERG* and * KCNJ15* exhibit strong linkage disequilibrium relationships with the significant genome-wide association SNPs identified in the second association signal

The second association peak included two genes, namely *ERG* and *KCNJ15*. As shown in Fig. 5, we observed a strong linkage disequilibrium between the leading associated SNP (Chr1:150739498) and other genome-wide significant SNPs, with an r^2^ value exceeding 0.6. The LD analysis revealed two genome-wide significant association SNP (Chr1:150612293 and Chr1:150612295) in *ERG* located in a 12 SNPs LD block (Table S5), while the two other genome-wide significant SNPs did not form an LD block (Figure S4). We identified that a stop-gained mutation (Chr1:150634326, c.C90G: p.Y30X) and a nonsynonymous mutation (Chr1:150561787, c.G1070C: p.R357P) in *ERG*. Additionally, we found that *KCNJ15* did not exhibit the significance associated with the genome-wide significant SNPs. These findings suggested that *ERG* is a potential candidate gene influencing the horned and polled phenotypes.

## Discussion

In this study, we performed a genome-wide association study (GWAS) using a high-density single nucleotide polymorphism (SNP) map obtained from whole-genome sequencing (WGS) to identify the genetic loci underlying the polled phenotype in Xinong Saanen dairy goats. Our analysis revealed 320 genome-wide significant SNPs located on chromosome 1, which formed two distinct association peaks consistent with previous GWAS findings for the polled phenotype. While our investigation showed slight variation in the range of these associated peaks compared to prior studies, the primary regions remained consistent, with both peaks centered around 129 Mb and 150.5 Mb [17]. These discrepancies may be attributed to variances in the detection platforms or algorithms employed during the corresponding analysis, alterations in the genetic background of the examined goats, disparities in the size and composition of the study population, or stochastic or technical errors encountered during certain analyses.

The most important problem in GWAS is the control of false positives for significant SNP [34]. In our study, there was a slight population stratification among the 106 individuals, which is a risk for the reliability of the results of GWAS. Currently, there exist four commonly used methods for addressing the issue of population stratification, namely genomic control [35], structured association analysis [36], PCA [36], and mixed linear models MLM [35]. This study chose MLM to control population stratification. The fixed effects in the model encompass genotypes and population structure while accounting for kinship as random effects [37]. This made the GWAS results more reliable.

Previous studies have reported that the absence of horn growth in goats is attributed to a complex structural genetic variant, characterized by the fusion of a large duplicated segment measuring 480 kb [38], located at position 150,334,286–150,818,098 bp on chromosome 1, into the deleted region spanning approximately 11.7 kb situated around 129 Mb on chromosome 1 [16, 17]. Despite the phenotypes, which are believed to result from an increase in gene copy number, often eluding explanation through changes in gene dosage [19]. The specific elements of these intricate structural variants that contribute to the absence of horn growth in goats remain unclear.

To further ascertain the causative variations underlying the two association signals, we performed the LD analysis. In the first associated genomic region, several SNPs and genes were identified as marker variants and potential candidate genes, respectively. For the first time, we identified 3 SNPs (Chr1:129789816, Chr1:129791507, and Chr1:129791577) that can be considered as potential indicators of the PIS-affected goats. These genome-wide significant SNPs have a strong LD relationship with gene *FOXL2. FOXL2* is widely recognized as a pivotal determinant of female sex development in mammals [33]. *FOXL2* was also identified as the headgear-specific expressed gene in bovids and cervids based on transcriptome analyses [39], indicating its potential involvement in horn growth. In addition, the genotype distributions at these loci are consistent with previous studies that there is the absence of intersexuality in homozygous polled goats with a male genotype (XY) and the inheritance of polledness in goats is predominantly associated with recessive intersexuality.

The LD block showed that there was a strong LD between gene *MRPS22* and 39 genome-wide significant association SNPs. The protein *MRPS22*, an essential component of the small subunit of mitochondrial ribosomes, plays a pivotal role in reproduction and ovarian development [40, 41]. According to the variant annotation, we identified two synonymous mutations and one nonsynonymous mutation (Chr1:129300519, G > A, p.A46V) within the exonic regions of *MRPS22*. Although these variants did not show significant associations in the genome-wide analysis, they formed an independent LD block with the 39 genome-wide markers specifically in horned goats. In contrast, no such association was observed in polled goats (including PIS goats). The present findings suggested that *MRPS22* may play a potential role in the development of infertile intersex individuals. Additionally, our findings have demonstrated that all 3 PIS goats exhibited homozygosity and carried variant alleles for the 39 significant genome-wide association SNPs in the first association region, whereas not all 33 horned goats displayed homozygosity and carried reference alleles. The genotype distributions at these loci exhibited an incomplete correlation with the horned, polled, and PIS phenotypes in the 106 Xinong Saanen dairy goats, suggesting that these 39 SNPs can not be considered as potential markers of the PIS-affected goats.

Another gene *MRAS* has 29 genome-wide significant association SNPs located in intronic regions. However, *MRAS* exhibited 4 synonymous mutations (Chr1:130287936, c.C > T, p.T198T, Chr1:130287942, c.C > A, p.R196R, Chr1:130296909, c.G > A, p.H102H, and Chr1:130296915, c.G > A, p.F100F) in the exonic regions. Therefore, these single nucleotide polymorphisms (SNPs) and the gene *MRAS* may not be considered as causative variations or candidate genes for the polled phenotype in goats. The remaining genes in the first associated region, namely *NMNAT3*, *RBP2*, *PIK3CB*, *ESYT3*, *SOX14*, and *PPP2R3A*, were not assigned to any LD block and their SNPs did not exhibit significant associations yet. Despite a previous study demonstrating the association of both *SOX14* and *ESYT3* with horn bud development in sheep [39, 42], our study findings do not support their role in the formation of thorned/polled phenotype in goats.

The second associated genomic region includes two genes, namely *ERG* and *KCNJ15.* The gene *ERG* is implicated in chromosomal translocations, resulting in the formation of distinct fusion gene products, such as TMPSSR2-ERG [43], which regulate the expressions of three osteoblastic markers (i.e., ET-1, COL1A1, and ALPL) at the cellular level [44]. The SNPs located within or near the *ERG* gene demonstrated significant associations with body conformation traits in cows [45]. Our LD analysis identified two genome-wide significant association SNPs (Chr1:150612293 and Chr1:150612295) in *ERG* that exhibit a strong linkage disequilibrium with 10 nearby SNPs, providing support for the potential regulatory roles of *ERG* in horn growth and metabolism in Xinong Saanen dairy goats. The gene *KCNJ15* is a protein-coding gene. The protein encoded by this gene functions as an integral membrane protein and exhibits characteristics of an inward-rectifier type potassium channel [46]. By analyzing the expression profile in otosclerosis patients, several studies have demonstrated the involvement of *KCNJ15* in aberrant bone remodeling processes in humans [47, 48]. However, our study revealed that the associations of SNPs in *KCNJ15* did not reach genome-wide significance.

The results presented in this study are based on theoretical causal variations derived from bioinformatics analysis. While these findings offer valuable insights into potential genetic markers for horn status and PIS, further experimental validation is necessary to confirm their functional significance. Future studies could focus on exploring the functional roles of these candidate genes through molecular biology techniques such as gene expression profiling or knockout experiments that could shed light on their precise contributions to horn development and PIS in goats. This work represents an important step towards unraveling the complex genetics behind horn status and PIS in goats. Three marker SNPs, which have been identified as closely linked to PIS, may contribute to the development of SNP chips for early prediction of PIS in goats. This advancement will facilitate breeding programs aimed at producing fertile herds with polled traits.

## Conclusion

In conclusion, our findings suggested the horned/polled phenotype of Xinong Saanen dairy goats is strongly associated with two regions located on chromosome 1. Furthermore, using GWAS and LD analyses, we successfully identified three marker SNPs that are closely linked to PIS and several candidate genes associated with polled traits in Xinong Saanen dairy goats. The findings could contribute to the breeding of fertile herds with polled traits.

### Electronic supplementary material

Below is the link to the electronic supplementary material.

Supplementary Material 1

Supplementary Material 2

## Data Availability

The requisite data for assessing the findings can be located within the manuscript and/or Supplementary Materials. The raw sequencing data of 106 Xinong Saanen dairy goats, comprising 33 horned individuals, 70 polled individuals, and 3 individuals with polled intersexuality syndrome, were deposited in the NCBI SRA database (accession: PRJNA1077730, https://www.ncbi.nlm.nih.gov/bioproject/PRJNA1077730). Any further inquiries should be directed to the corresponding author.

## Abbreviations

GWAS: Genome-wide association study
LD: Linkage disequilibrium
MLM: Mixed linear model
PCA: Principal component analysis
PIS: Polled intersexuality syndrome
SNP: Single nucleotide polymorphism
WGS: Whole genome sequencing
