# Supplementary material for: Genome-wide association study provided insights into the polled phenotype and polled intersex syndrome (PIS) in goats
Source: BMC Genomics. 2024 Jul 2;25:661. doi: 10.1186/s12864-024-10568-9 (PMC11218382; doi:10.1186/s12864-024-10568-9)
Supplement: Supplementary file 2 — Supplementary Material 2 [file 12864_2024_10568_MOESM2_ESM.docx]

Supplementary Materials for

**Genome-wide association study provided insights into the polled phenotype and polled intersex syndrome (PIS) in goats**

Fuhong Zhang *et al.*

*Corresponding author. Email: [luojun@nwafu.edu.cn](javascript:;)

**This PDF file includes:**

Supplementary Text

Figures. S1 to S4

Tables. S1 to S5

**Supplementary Figures**


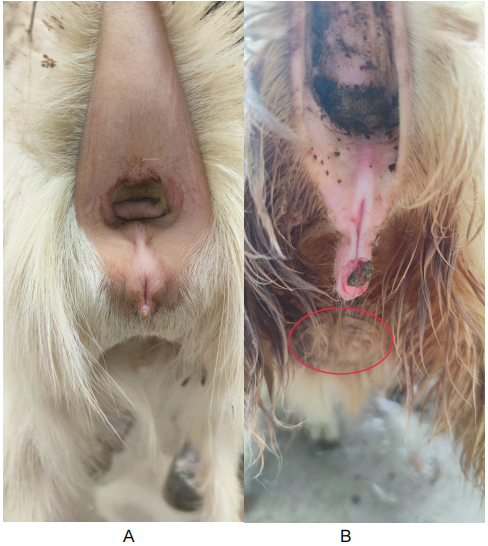


Figure S1. Comparison of the external genitalia of a normal (A) and PIS-affected (B) polled Saanen goat. PIS, polled intersexuality syndrome. Testicles are circled in red.


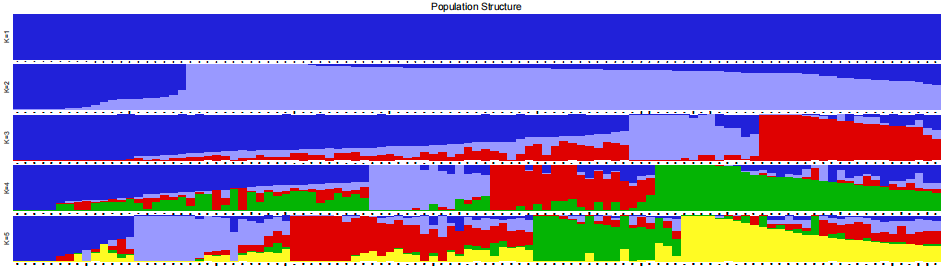


Figure S2. The population structure of 106 sampled goats based on the identified biallelic SNPs.


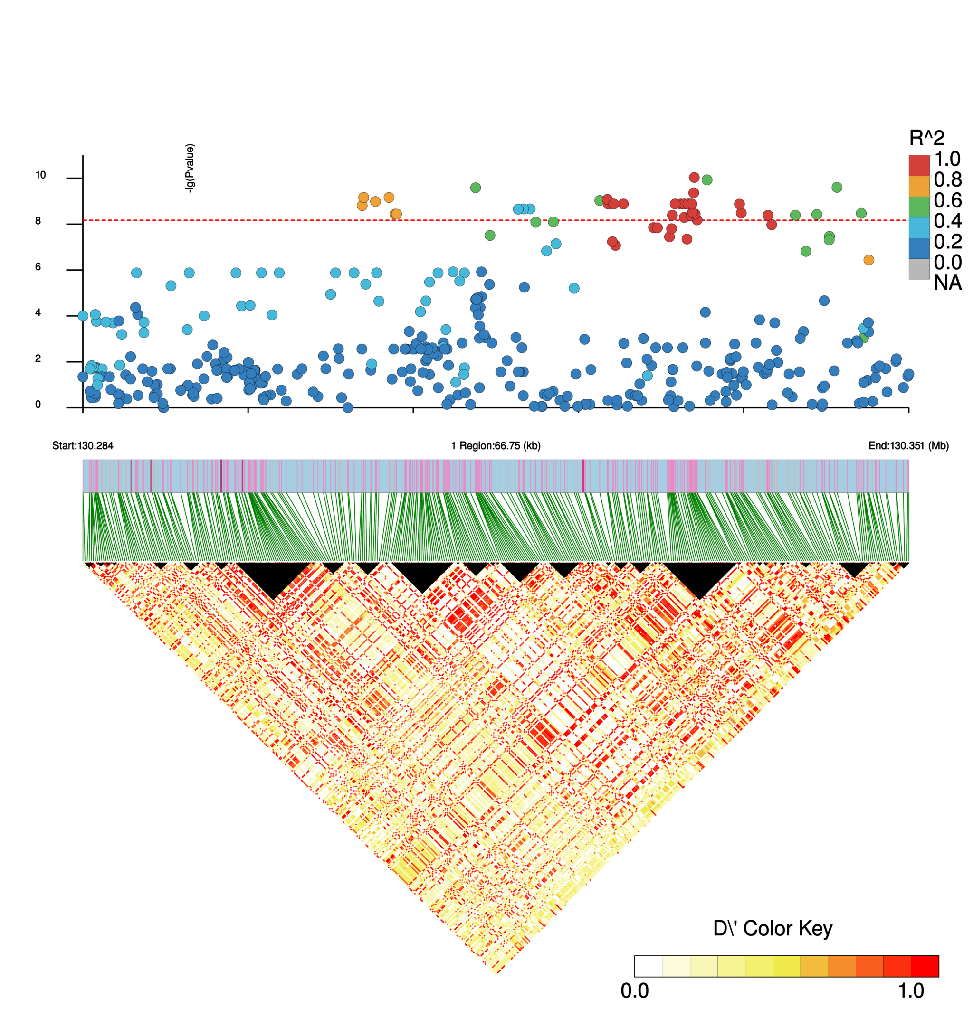


Figure S3. The LD block analysis for 29 genome-wide significant association SNPs located in intronic regions of *MRAS.*


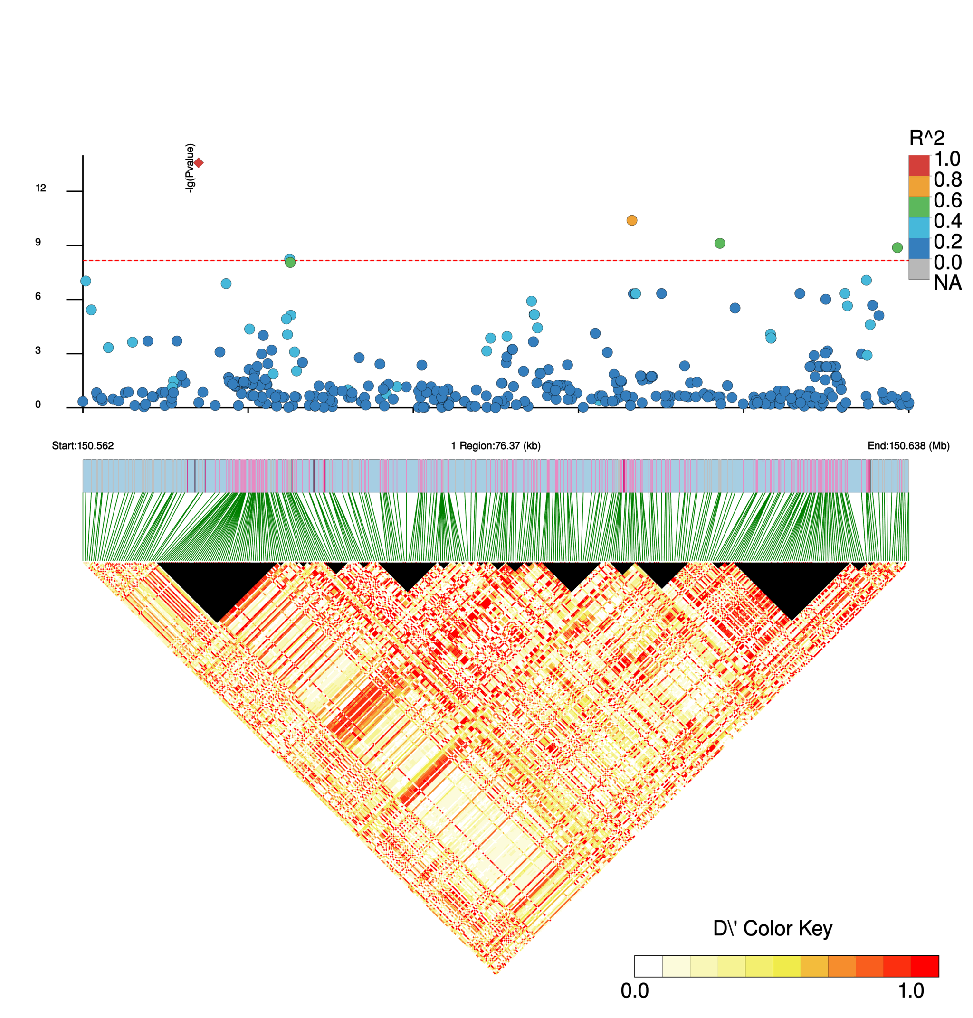


Figure S4.The LD block analysis for 4 genome-wide significant association SNPs located in intronic regions of *ERG.*

**Supplementary Tables**

**Table S1. Statistics of gene families cluster analysis**

| Samples | Famale/Male/PIS | Horned/Polled | N_Sites | Mean_Depth |
| --- | --- | --- | --- | --- |
| XN100A | Male | Polled | 31721184 | 15.4387 |
| XN101A | Male | Horned | 31721480 | 15.2359 |
| XN102A | Male | Polled | 31721541 | 15.3569 |
| XN103A | Female | Horned | 31721568 | 16.8875 |
| XN104A | Male | Polled | 31722180 | 22.3462 |
| XN105A | Female | Polled | 31721447 | 13.4786 |
| XN106A | Female | Polled | 31721821 | 15.2582 |
| XN107A | Female | Polled | 31721046 | 12.1977 |
| XN108A | Female | Polled | 31721686 | 14.8454 |
| XN109A | Female | Polled | 31721508 | 13.6669 |
| XN11A | Male | Polled | 31720754 | 11.6061 |
| XN12A | Female | Polled | 31720958 | 12.1249 |
| XN13A | Male | Polled | 31721006 | 14.7815 |
| XN14A | Male | Polled | 31721071 | 11.081 |
| XN15A | Male | Polled | 31721589 | 16.1407 |
| XN16A | Female | Horned | 31719969 | 9.02975 |
| XN17A | Female | Polled | 31720858 | 10.9824 |
| XN18A | Female | Polled | 31720486 | 10.8546 |
| XN19A | Female | Polled | 31721118 | 12.9606 |
| XN1A | Male | Horned | 31721792 | 15.9374 |
| XN20A | Male | Polled | 31720620 | 11.6047 |
| XN21A | Male | Polled | 31721091 | 14.2305 |
| XN22A | Male | Polled | 31720917 | 12.3435 |
| XN23A | Male | Polled | 31721318 | 13.298 |
| XN24A | Male | Polled | 31721103 | 14.6419 |
| XN25A | Male | Horned | 31720423 | 11.4266 |
| XN26A | Male | Polled | 31721449 | 14.3536 |
| XN27A | Female | Polled | 31721052 | 12.5085 |
| XN28A | Female | Horned | 31721238 | 11.8009 |
| XN29A | Female | Horned | 31721161 | 12.3187 |
| XN2A | Male | Polled | 31721740 | 16.5702 |
| XN30A | Female | Horned | 31721308 | 13.6768 |
| XN31A | Female | Polled | 31720352 | 9.10408 |
| XN32A | Female | Horned | 31721382 | 13.954 |
| XN33A | Female | Polled | 31721544 | 14.2092 |
| XN34A | Female | Polled | 31721493 | 13.282 |
| XN35A | Female | Polled | 31721101 | 12.9671 |
| XN36A | Female | Horned | 31721796 | 16.6711 |
| XN37A | Female | Polled | 31721304 | 13.2381 |
| XN38A | Female | Polled | 31720335 | 10.1809 |
| XN39A | Female | Horned | 31720505 | 11.5739 |
| XN40A | Female | Polled | 31720376 | 9.49221 |
| XN41A | Female | Polled | 31720539 | 9.82948 |
| XN42A | Female | Polled | 31720584 | 10.8913 |
| XN43A | Female | Polled | 31720439 | 9.70161 |
| XN44A | Female | Polled | 31720699 | 10.9311 |
| XN45A | Female | Polled | 31720490 | 10.4946 |
| XN46A | Female | Polled | 31720996 | 11.7446 |
| XN47A | Female | Horned | 31721480 | 13.6943 |
| XN48A | Female | Horned | 31720563 | 10.9475 |
| XN49A | Female | Horned | 31719939 | 8.99085 |
| XN50A | Female | Polled | 31720560 | 10.8384 |
| XN51A | Female | Polled | 31719976 | 8.9232 |
| XN52A | Female | Polled | 31720519 | 9.51209 |
| XN53A | Female | Polled | 31721211 | 12.5306 |
| XN54A | Female | Polled | 31720527 | 10.7168 |
| XN55A | Female | Horned | 31720943 | 11.138 |
| XN56A | Female | Polled | 31721230 | 12.341 |
| XN57A | Female | Horned | 31721011 | 11.5327 |
| XN58A | Female | Horned | 31720708 | 11.4169 |
| XN59A | Female | Polled | 31720331 | 9.16252 |
| XN60A | Female | Horned | 31721056 | 11.8185 |
| XN61A | Female | Horned | 31720307 | 9.69521 |
| XN62A | Female | Polled | 31721180 | 12.5133 |
| XN63A | Female | Polled | 31720545 | 10.3989 |
| XN64A | Male | Polled | 31720979 | 12.0779 |
| XN65A | Female | Polled | 31721328 | 14.416 |
| XN66A | Female | Horned | 31720728 | 10.9257 |
| XN67A | Female | Polled | 31721239 | 12.3433 |
| XN68A | Female | Horned | 31721617 | 15.1973 |
| XN69A | Female | Polled | 31721528 | 13.417 |
| XN6A | Female | Horned | 31721472 | 16.2926 |
| XN70A | Male | Horned | 31721111 | 11.9572 |
| XN71A | Male | Polled | 31719538 | 11.7591 |
| XN72A | Female | Horned | 31720237 | 12.2876 |
| XN73A | Male | Horned | 31721148 | 12.7016 |
| XN74A | Male | Polled | 31721069 | 13.0054 |
| XN75A | Male | Horned | 31720094 | 9.57302 |
| XN76A | Male | Polled | 31721552 | 13.7901 |
| XN77A | Female | Horned | 31721040 | 15.6116 |
| XN78A | Female | Polled | 31721219 | 12.5452 |
| XN79A | Female | Horned | 31721368 | 14.6025 |
| XN80A | Male | Polled | 31721612 | 14.6759 |
| XN81A | Male | Polled | 31721204 | 13.4952 |
| XN82A | Female | Polled | 31720968 | 12.0132 |
| XN83A | Female | Polled | 31721184 | 12.441 |
| XN84A | Male | Polled | 31721475 | 14.1863 |
| XN85A | Female | Polled | 31721033 | 13.5575 |
| XN86A | Male | Horned | 31721640 | 14.7463 |
| XN87A | Female | Polled | 31721158 | 12.7466 |
| XN88A | Male | Horned | 31721192 | 14.3223 |
| XN89A | Female | Polled | 31721538 | 18.8938 |
| XN90A | Male | Polled | 31720991 | 12.0303 |
| XN91A | Female | Polled | 31721712 | 16.711 |
| XN92A | Male | Polled | 31721100 | 15.5023 |
| XN93A | Male | Horned | 31721059 | 15.9594 |
| XN94A | Male | Polled | 31721766 | 15.7189 |
| XN95A | Male | Polled | 31721246 | 14.5844 |
| XN96A | Male | Horned | 31721924 | 23.0149 |
| XN97A | Male | Polled | 31721623 | 15.6853 |
| XN98A | Male | Polled | 31721955 | 18.371 |
| XN99A | Male | Polled | 31721487 | 16.0851 |
| XN9A | Female | Horned | 31721905 | 17.9987 |
| XNJX01A | PIS | Polled | 31721485 | 14.7908 |
| XNJX02A | PIS | Polled | 31720635 | 10.6646 |
| XNJX03A | PIS | Polled | 31721262 | 14.0995 |

**Table** **S2. The SNPs significantly associated with the horned and polled phenotypes.**

(Excel table)

**Table S3. The identified SNPs were annotated using the ANNOVAR software.**

(Excel table)

**Table S4. The results of LD block analysis using LDBlockShow.**

(Excel table)

**Table S5. The results of LD block analysis using LDBlockShow.**

(Excel table)
